# Life and Labour
*The Effects of Arts, Trades, and Professions, on Health and Longevity. By C. T. Thackrah, Esq. London: 1832.


**Published:** 1859-01

**Authors:** 


### SANITARY REVIEW.

JANUARY 1859.

### LIFE AND LABOUR.*

If the results of the labours of a modern statistical inquirer, Dr. Guy, have indicated by figures the truth of the saying, that man shall live by the sweat of his brow ; if it be true, as without doubt it is, that the life is both short and heavy into which labour is not infused,—it is not the less true that the reverse of the rule is in our days a prominent fact, and that labour is the parent of a huge mortality.

It requires an intimate knowledge of the habits and life of the poorer classes, and of the practical working of the businesses upon which these classes depend for subsistence, before even an approach can be made towards the fact of the relationship which exists between occupation and mortality. In some measure the statistics of mere mortality-registers give, it is true, an approximation towards the fact. They shew that some persons of such and such an occupation die of this or that disease,—of phthisis on this hand, or of nervous disease on the other. In this light, statistics of mortality are of great value ; yet of a value limited in degree. In them the outward and general expression of a hidden evil is supplied : they are as the index of a book, which gives the names of the subjects considered, but offers none of the argument, and cannot be said even to condense the information.

To arrive at the truth, to know to what extent life and health are sacrificed—and often needlessly sacrificed—to the Moloch toil, the working philanthropist must leave the statist's quarry, and out in the world at large learn for himself, amidst the teeming and sweating millions, how they suffer and how they die, not at, but from, their tasks, and by them. For the fact seems, that those engaged at various occupations are often themselves unaware that the business, by which they are earning their bread, is the guide to their deathbeds. Worn

* The Effects of Arts, Trades, and Professions, on Health and Longevity. By C. T. Thackrah, Esq. London: 1832.

by some chronic malady which disables them from work, they pine out the remainder of their days in the rest enforced by their complaint; or the suffering to which they were first subjected solely from labour becomes, in the end, modified in character, and assumes in disease a new name. Hence, dying at a late period after the last day's work, and from what seems, and often is, a new and acute phase in disease, the death is chronicled in a way which may not in any manner connect the death with its one and primitive cause.

In instituting a rigid inquiry into the question of the influence of occupation on mortality, great difficulties are met with. The evil effects of occupation (often, as we have said, unknown) are still more frequently known, and hidden. The employers of artisans engaged in injurious arts—we mean injurious to the artisans—are not, as a general rule, over-sensitive to the comfort and the safety of the employed; nor are they ready, or at any time willing, to communicate truths which tell against their own plans and business arrangements. The employed, again, even when conscious that they are enduring a daily cross, are timid in giving satisfactory information, which may compromise them with their taskmasters. Or, again, if they are willing to communicate, they are led unintentionally to misrepresentation, like all who are not educated in the art of observation.

The only means that could be taken to expose the dangers of injurious occupations, and to remedy evils which every day seem on the increase, would be, to set to work the machinery of a royal commission of inquiry. Such commission, formed of intelligent men, might well sit for several sessions at their required work. The result of their labours would, we submit, unfold a tale which would make the philanthropic world shudder, and open to it a field of industry regarding which it is, at the present moment, practically ignorant, and towards which it is, of consequence, practically supine.

The only men who are, at this moment, fully alive to the evils of which we write, are the members of the medical profession; and even amongst these there is an apathy from which we would endeavour respectfully to awaken them. We find, in our medical literature, much matter of interest in regard to various causes of disease, but very little in the way of reference to occupation as one of the most common causes of innumerable maladies. Yet we know that members of the medical body have immense stores of knowledge on the subject in hand; and we know that their sympathies are ever kept awakened by the histories of disease and labour which they are constantly forced to hear. Let us then earnestly summon our great profession to a new work, or rather to a public work; to an exposition made to the world at large, of medical facts on a question of national interest.

The work has, indeed, been briefly opened and reopened for discussion. The book noticed at the commencement of our paper is an instance. Thackrah—a man, if not of genius, certainly of great talent, and of a noble philanthropy—did all he could, with the means he had at command, to awaken public attention to the artisan and the artisan's imposed maladies. Within the last few years, too, the Society of Arts founded a committee for inquiring into "industrial pathology"; but as yet the labours of the committee have borne little fruit; and Thackrah's well meant and able teaching has had but trifling influence on the people at large.

In our own sphere of observation we have of late years been endeavouring to collate certain facts and observations on the subject under consideration. The facts, up to this time, are limited in number, and the space for their communication is necessarily limited also. But such evidence as we do communicate is reliable; and may, to say the least of it, draw the minds of many readers to similar lines of research.

The manufacture of *Sand-Paper* is an occupation which, as we opine, is attended with serious evils. In this occupation the pulverised sand, or pulverised sand and glass, is placed in fine sieves; and, by gentle motion, the powder is equally distributed over paper prepared for its reception. The distribution is done by the hand; and as it is light work, young boys, hardly relieved from leading strings, are employed at it. The result is, that the distributor, from the moment his work commences, is exposed to the inhalation of the irritative dust. A boy was one day brought before us, fatally struck by a chest affection resembling acute pulmonary consumption. On inquiry, we found that the youth, then engaged at the sand-paper business, was in perfect health until the first day he entered on his work. On his return in the evening to his home, he brought with him cough and thoracic pain, which never left him. Quickly the health failed; and when we saw him only three weeks after the commencement of the labour, his chances of life were over. He died within a month. Making further inquiry into this business, we found that this poor youth was the representative of a great class of youths similarly disabled. There were few who ever stood the work. It adds to the shame of these occurrences, that, except in the matter of pecuniary saving in the purchase of young hands, all risk is unnecessary; that by very simple machinery the manufacturer might dispense with the distribution by hand altogether; and that the particles of irritative powder need never be so distributed through the air as to be breathed either by the manufacturers or their assistants.

*Walking-Stick Making* is another business which carries with it mischiefs little less serious than the above. The sufferers, however, in this case are men; and the mischiefs induced, as described with great exactness and common sense by one of the sufferers, occur as follow. In order to make common sticks assume various colours and shades, the sticks, when turned and ready for colouring, are gently charred over a coke fire. The men engaged in this department suffer severely from the coke fumes. They grow giddy, lose appetite, and are altogether greatly enfeebled. But more serious danger resides in another stage of the process. The stick having been charred externally, the shading begins. The art of this lies in removing, by a fine rasp and by sand-paper, proper amounts of the charred material. An atmosphere charged with pulverised charcoal is thus produced; and the irritative effect of such inhalation on the lungs of the man, begins almost from the first day of the employment. In the man who came under our notice, the symptoms which had long been present were bronchial, but the mischief was so extensive that his life was clearly shortened by it, and a malady never to be removed was on him. He had constant cough, a flux from the lungs charged with the charcoal particles, and great emaciation. By removing from his work on our suggestion, this man made a partial recovery, and is, we believe, still alive.

From such information as we could gather from this man, we learned that the cough incident to the occupation is attended in the first instance with considerable acute suffering; but that by and bye, as the cough loosens, and as a steady expectoration of secretion charged with the sooty matter takes place, the acute symptoms give way to chronic cough and bronchial flux. "It is all right", said our informant, in referring to the charcoal dust—"it is all right when we can cough it up; if a man get into that way he stands his work for many years; but eventually, the cough always masters us, and so we break down."

The remedy against this evil lies probably with the men themselves. We suggested to our informant that a woollen muffler would be a capital filter and protect effectually. "Very simple, sir," was the reply, "but quite impracticable, 'specially in hot weather, when we feel the dust most."

*Hemp and Flax-Dressing* are occupations attended inevitably, as all evidence goes to show, by a series of symptoms, affecting chiefly the bronchial surface, which may be considered specific in kind. We were fortunate, in our inquiries, in having the opportunity of examining an intelligent workman who had been engaged at the business of hemp-dressing and flax-dressing for thirty years; in this time, he had left his work during certain intervals only, in which he had been sick-bound. This man, from the first a sufferer from his occupation, had come into contact, at various times, with many hundreds of men in the same line of life, and he was thus enabled to give us some very correct and extended information. He was quite unbiassed in his narrative; he had great respect for his employers; and so conscientious was he in his statements, that he paid us a special call to correct a slight error into which he thought he had fallen. The experience of this witness is confirmed by that of two others, engaged in the same business at other manufacturers. The history of the dressing process is somewhat as follows.

The raw hemp comes mainly from Russia and Poland. These countries send the finer qualities of hemp. But a coarser variety is imported from Naples, and this variety was largely used during the late war. The first step in the process of dressing, consists in drawing the rough hemp through the "hackle," a machine in large manufactories consisting of benches in which are inserted series of pins close together, in the manner of a comb of many rows. The hemp, taken by lengths weighing about a pound, is drawn first by an overhand movement, through a coarse set of the pins, and then by an underhand movement through a finer series. These acts complete the primary steps of the process, and during these steps the men suffer very severely; during the whole time the air is charged with dust and fluff. Some idea of the extent to which the fluff is dispersed, may be gleaned from the fact, that, of every hundred and twelve pounds weight of hemp employed, there is escape and loss of four pounds in the dressing. The Neapolitan hemp is much the most indifferent in this respect.

The man to whom we referred at the opening of this section was a sufferer from the first, from his employment. He never could become tolerant of it; and at no time could remain longer than three hours at his work, without going out of doors to restore himself with fresh air. He was subject to constant irritating cough and constant expectoration, very profuse in amount. In the course of his labours, he assured us, he had never known a fellow-workman who did not suffer from the same affection. In some, however, the cough was dry and husky, and even more trying. In country places the workmen suffer less than in London, and in London the work is much more tolerable in warm and dry weather. During fogs, when the fluff cannot find escape freely by currents of air, and when the air itself is of difficult respiration, the labour is hardly to be borne.

There is another curious fact connected with the hemp business which deserves notice. From the Neapolitan hemp there emanates, spontaneously, an odour of a very irritative character; the inhalation of which causes shortness of breath, constriction of the throat, and spasmodic cough in paroxysms. All workmen with this variety of hemp seem susceptible. To satisfy ourselves on this subject, we obtained a portion of Neapolitan hemp which was assumed to have this quality, and soon experienced in our own proper person the truth of the allegations against it. The symptoms caused by it are those of "hay fever," or like the symptoms produced in some persons by ipecacuan. Nor does the dressing of this hemp remove the mischief; for the symptoms are common to the spinners of the dressed variety, although, from working in the air, these are much less exposed to the fluff.

*Flax-Dressing*, which is, in fact, but a continuance of the above process, on a finer kind of hemp, is equally injurious to the workmen, and is even worse, as an occupation, than the dressing of Russian hemp. The loss of fluff in flax-work is nearly the same as in the preparation of hemp.

It is possible that a certain amount of mischief to the lungs must always follow the hemp and flax manufactures. But much of the evil might be avoided by a better ventilation in the manufactory. Ordinarily, in the work-rooms, no effort whatever is made to ventilate, and the fluff deposits in abundance on the floor, whence it is again distributed through the air on the merest movement. The spinners of hemp who work in the open air, are only subject to the special ailment caused by the Neapolitan hemp.

*Trimming Manufacture*, in which the fluff of silken fabrics is freely distributed through the workroom, leads to symptoms of a chronic bronchial kind, not unlike, but perhaps less in degree than, those caused by the hemp fluff. A woman from one of these manufactories came before us with profuse expectoration and cough. The expectoration was coloured as if with blood, and this circumstance had alarmed her considerably. On inquiry; we found that the colour imparted to the secretion was really not due to blood, but to some substance of fibrous texture and red tint. We should have thought that the patient was feigning, but for the physical and unmistakeable evidence of severe bronchial disorder. The explanation of her occupation on the part of the patient, cleared up all the difficulty. The woman had been engaged in working red silk, and exposed to the silk fluff, which is given off in great quantities during the twisting process in the making of tassels. The sister of this same person worked at the same business, and suffered in the same way. We found, ultimately, that in the small unventilated room in which this woman spent her working hours with her sister, there were three more occupants, engaged in the same pursuit, and paying the same penalty. The business is one which few can bear for more than a few years, while some break down at once. Much depends on the size and ventilation of the workroom.

We cannot here omit noticing a remark made to us by a woman to whom our inquiries were being directed, in relation to the accommodations of women in workrooms and manufactories. We always "feel the room," said this person, "more than men, because we are put closer together." Why? we asked. "I do not know," was the reply, "but it seems to be an opinion amongst the masters that women can do with less room and put up with fewer accommodations than men." Like our witness, we cannot say why this is the fact; but on better acquaintance with the facts under consideration, we can confirm every word of her remark. Go into a work-room in which ten men would consider themselves half suffocated; change the occupants for women, instead of men, and sure as fate, where the ten men were arranged, fifteen women will be packed. Such perversity is unaccountable, idiotic we may say. But still the fact, still the fact.

*Fur-Dyeing* is another art, the evils of which to the artisan are many, and as serious as manifold. In the young and feeble workers, phthisis is the prevailing malady, in the more advanced chronic bronchitis in its most persistent form. These are the maladies. Let us but sketch out the nature of this occupation, and the mischief of it will not be far behind in the mind.

The object of the fur-dyeing process is threefold :—

1. To make bad furs look perfect.
2. To make the fur of one animal resemble that of another and different animal.
3. To change the colour of various furs.

The materials employed by the fur-dyer for the completion of one or other of these processes are—nitric acid, sal ammoniac, liquid ammonia, antimony, verdigris, litharge, alum, copper, copperas, lime, pearlash, soda, and gall-nuts. The nitric acid is used as a cleansing substance, and also as a staining material to impart a yellow tint to some skins and to lighten the colour of skins which are black. The sal ammoniac, lime, litharge, and soda, mixed up together into solution with water, are employed to remove grease from skins that are going to be turned into a dark colour. The skins are brushed over with this solution.

The colouring fluids used are, first, as we have said, nitric acid. Secondly, a solution made as follows: Copperas, one pound; water twelve pints. This solution is used for producing a very black dye. Thirdly, a solution made thus: Gall-nuts roasted and ground into a powder, one pound; copperas, three ounces; sal ammoniac, two ounces; verdigris, two ounces and a half; litharge, one ounce and a half; copper-dust, two ounces; sumach, three ounces; these are all mixed together with water, so as to make in the whole three quarts of solution. This colouring solution gives either a light or a dark brown dye; light if much water is used, or if only one dressing is given; dark if the solution is concentrated, or if the fur is many times washed over with it.

When a fur is going to be coloured, it is washed over either with nitric acid or with the solution of sal ammoniac, lime, litharge, and soda, named above. The skin is then dried and the dust is beaten out of it; then the colouring solution, whichever it may be, is applied with a stiff brush, time after time, until the desired shade is obtained.

For the colouring of grenadier's bearskin caps, gall-nuts are boiled with the solution to be employed. The grenadier's cap is soaked in the solution.

A variety of other solutions have been tried in the fur-dyeing business, such as indigo, muriate of tin, and muriate of iron. They all fail. The muriate of iron colours the fur, but destroys the skin.

The injuries to health arising from this business occur at different stages of the process. In the first step, where the solution of nitric acid is being applied, a curious set of symptoms appear. As the nitrous acid fumes are inhaled, the mouth, tongue, and fauces are rendered dry and irritable, there is constant constipation, there is constant headache, and the pain in the head is invariably situated in the back part. Later, the skins, moist with the acid solution, are placed (it is the fact) in an air-tight heated chamber to *dry*. When the door of this chamber is opened, there is a perfect flood of acid vapour set free, in which the workmen are bathed. The effect of the inhalation is invariably to cause cough, dryness of the mouth and throat, constriction of the chest, and pain in the head.

When, instead of nitric acid, the second solution described above is used for the removal of greasy matter from the skins, ammoniacal fumes rise and excite a continued hacking cough, a languor and a headache which can hardly be borne; a stranger to the work cannot tolerate it at all. The drying, after the application of this solution, is done as before, in a large air-tight chamber heated to 130° or more. The workmen have often to enter this room and remain in it several minutes for the purpose of removing dried skins and turning drying ones. The effort against absolute suffocation is tremendous during the performance of this duty.

It is but right to add that in some manufactories, as at Appold's, the principles of common sense in drying have prevailed, and that a ventilating shaft has been introduced into the heated chamber, through which the gases can escape. In smaller manufactories, no such provision is made.

A third and serious mischief is produced by the inhalation of the dust during the beating of the dried skins which have been previously soaked in the copperas solution. The dust thus discharged produces constant irritating cough, and the teeth are almost invariably affected by it; these organs are slowly rendered brittle and generally carious. The grinding down of the colour stuffs from the crystalline to the pulverulent state leads to similar evil consequences. Thus in every part of this business the workmen inevitably suffer.

The remedies of a preventive kind in this occupation are obvious. The exposure to the nitrous acid fumes is removable by free ventilation. The dusting process could all be done by machinery similar to that used for common threshing purposes, and the grinding of the powder by mill-work. There is no class of occupation requiring more urgent reform than the fur-dyeing manufacture.

*Cigar and Snuff Making* are businesses accompanied by results little less injurious than those arising from the afore-named pursuits. The manufacture of cigars consists in rolling up the leaves of the tobacco-plant into the cigar shape; or, in inferior cigars, in rolling up the leaf over a quantity of broken up tobacco-leaf together with siftings, and even with the sweepings of the shop. The unanimous testimony of several workmen whom we have examined is to the effect, that, in the most common cigar, tobacco-leaf is always used. There is no other leaf, such as cabbage-leaf, used in the process.

At a further stage of the process, the cigar is placed in the drying-room. During drying the room is charged with vapour, which has much the same effect on the body as tobacco-smoke.

In making snuff, tobacco-leaf is finely cut up, well dried, and then pulverised together with an incongruous collection of foreign matter: lime, salt, flour, dust-sweepings, and, in yellow snuffs, lead; or, as one workman remarked, "anything else, as everything does for snuff that will powder fine." The mixture once made, it is placed in a bin, and heat is applied. Two or three times the mixture is removed from the bin to be turned. The heating process gives a sharpness to the snuff.

When the sharpening experiment is concluded, the mixture is sent to the mills to be ground and well dried. Then it is sifted; and lastly, the "liquor" is added to make the powder weigh heavier. The liquor is a solution of common salt and water. The hotch-potch is now finished, and is ready for the nose of the unfortunate consuming sufferer, whom may it continue to please.

The evils to the workmen abound. The rooms in which they work are close, unventilated, and filled with dust, which inhaled, gives rise to throat irritation, cough, and vomiting. Sifting the shorts is in this way more hurtful than rolling the dust cigar. The inhalation of the vapour from the cigar drying-room sets up in young hands all the symptoms of tobacco poisoning: dryness of throat, cough, headache, and vomiting are the prominent symptoms. In snuff-making, the turning of the mixture which is being heated in the bin sets up the poison symptoms even more effectually. The young workman at this department is seriously poisoned; he grows faint and sick, and suffers from violent head-symptoms. His consolation is that he shall get used to the poison if he only sticks to it; and, as far as acute symptoms are concerned, he is not, as a general rule, deceived. The sifting of the dried and ground snuff is followed by analogous effects.

But it is not to be presumed that the disease symptoms stop where we have left them above. The system of the workman becomes tolerant in some measure; but the tolerance is only partial. There are induced, by continued application, chronic maladies which do not so easily desert him, and which sometimes cost him his life. Pulmonary consumption is one of these diseases. In less serious cases, dyspepsia, anæmia, and rapid and irregular action of the heart present themselves as symptoms, and continue so long as the occupation is the means of obtaining that daily bread which is purchased to ply a poisoned body.

We must not omit to mention, that the occupation leads to excessive abuse in the habits of smoking and chewing tobacco. One man informed us that some workmen seemed as if they could not exist unless they were smoking or chewing.

From these combined evils few workmen, if any, escape; they are rarely able to follow their employment beyond the age of two score years.

In relation to the mischiefs due to the cigar and snuff manufactures, many philanthropists would doubtless offer a sweeping recipe. They would do away with the evil wholesale. We, taking a more practical view, and feeling that any attempt to suppress the business altogether is untenable, would simply suggest palliative measures. These would consist in securing a thorough ventilation of the work-rooms, a plan for turning over the drying snuff with the mouth and nostrils of the turners protected, and the sifting of the snuff by machinery worked in closed boxes or small chambers.

We have thus sketched out six illustrations of life sacrificed to labour. We must not in one number trespass with further instances. The reader who has followed us so far will have observed, that all the examples supplied of diseases arising from occupations, are examples in which the lungs are prominently and mainly affected. If we were to follow out the inquiry to its fullest extent, we believe that the same general rule would be found to prevail.

There are truly some disorders which are localised in other than the lung-structures, and which result from occupation. In this category we may name the local affection of the jaw arising from phosphorus; the curvature of spine incident to long confined position of body; the rheumatic affection of joint arising from labours conducted in underground caverns, and which expose the men to constant damp; and so on, through, it may be, twenty exceptional cases. But the grand rule obtains, we say, in the majority of occupations, that the lungs are the seat of the induced disease. When air is inhaled rendered simply impure by vitiation with the air expired by the subject, the symptoms are chronic in character and assume the phthisical type. When the air is surcharged with dust or fluff, the irritating agent applied directly to the minute ramifications of the air-tubes sets up bronchial mischief, and either at once destroys by a series of exhaustive symptoms resembling phthisis, or leads to chronic disease and a persistent bronchial flux.

In other classes of cases, where the foreign body inhaled is not only irritating to the lungs, but is capable of entering the body by absorption from the lungs into the blood, as in the manufacture of snuff and in the drying of cigars, general symptoms, specific to the poison inhaled, are the result.

When we look at diseases as a whole, we stand amazed at the varieties of type which they assume. When we classify them into groups according to their causes, we stand equally amazed at finding to how very few groups all diseases may be reduced. We find all the disease-causes out of the body, and clothed in externals. Resolving the causes, there stand out some dozen poisons of communicable and reproductive power, as improper dietary, variations of atmosphere, and occupation. Of all these, the last stands most invitingly for inquiry. The inquiry is of a kind to which the most rigid rules are applicable. Its results might be demonstrations; its suggested remedies simple certainties. Surely greater inducements to the performance of a great national requirement never before presented themselves to the ministerial mind.

---

